# Single-Shot ChAd3-MARV Vaccine in Modified Formulation Buffer Shows 100% Protection of NHPs

**DOI:** 10.3390/vaccines10111935

**Published:** 2022-11-15

**Authors:** Courtney L. Finch, Thomas H. King, Kendra J. Alfson, Katie A. Albanese, Julianne N. P. Smith, Paul Smock, Jocelyn Jakubik, Yenny Goez-Gazi, Michal Gazi, John W. Dutton, Elizabeth A. Clemmons, Marc E. Mattix, Ricardo Carrion, Thomas Rudge, Alex Ridenour, Sovann F. Woodin, Ruth Hunegnaw, Nancy J. Sullivan, Rong Xu

**Affiliations:** 1 Sabin Vaccine Institute, Washington, DC 20037, USA; 2 Texas Biomedical Research Institute, San Antonio, TX 78227, USA; 3 Battelle Biomedical Research Center, Madison County, OH 43162, USA; 4 Nonclinical Pathology Services, LLC, Medina, OH 44256, USA; 5 Vaccine Research Center, National Institute of Allergy and Infectious Diseases, National Institutes of Health, Bethesda, MD 20892, USA; 6 Clover Biopharmaceuticals, Boston, MA 02109, USA

**Keywords:** Marburg virus, adenovirus, vaccine, glycoprotein, filovirus, nonhuman primate, cynomolgus macaque, crab-eating macaque

## Abstract

Marburg virus (MARV) is a virus of high human consequence with a case fatality rate of 24–88%. The global health and national security risks posed by Marburg virus disease (MVD) underscore the compelling need for a prophylactic vaccine, but no candidate has yet reached regulatory approval. Here, we evaluate a replication-defective chimpanzee adenovirus type 3 (ChAd3)-vectored MARV Angola glycoprotein (GP)-expressing vaccine against lethal MARV challenge in macaques. The ChAd3 platform has previously been reported to protect against the MARV-related viruses, Ebola virus (EBOV) and Sudan virus (SUDV), and MARV itself in macaques, with immunogenicity demonstrated in macaques and humans. In this study, we present data showing 100% protection against MARV Angola challenge (versus 0% control survival) and associated production of GP-specific IgGs generated by the ChAd3-MARV vaccine following a single dose of 1 × 10^11^ virus particles prepared in a new clinical formulation buffer designed to enhance product stability. These results are consistent with previously described data using the same vaccine in a different formulation and laboratory, demonstrating the reproducible and robust protective efficacy elicited by this promising vaccine for the prevention of MVD. Additionally, a qualified anti-GP MARV IgG ELISA was developed as a critical pre-requisite for clinical advancement and regulatory approval.

## 1. Introduction

Marburg virus (MARV), a filovirus, is the causative agent of Marburg virus disease (MVD), a high consequence public health threat with a case fatality rate of 24–88% [1]. After an incubation period that is typical for the filovirus family (as long as 21 days but generally not longer than 10 days), disease presentation is first characterized by non-specific signs and symptoms such as fever, myalgia, headache and chills which can progress to nausea, diarrhea, cramping and vomiting [1,2,3]. Infection spreads systemically, particularly to the liver, spleen and lymph nodes, and hemorrhaging from multiple mucosal sites may occur [1,2,3].

MARV is endemic to the African continent. Outbreaks have been documented in Uganda, Kenya, Democratic Republic of the Congo and South Africa (as well as outside of Africa). Recurrent outbreaks have been documented in Uganda and Kenya [1]. Generally, outbreaks occur infrequently and in small numbers but with great loss of human life accompanied by social and economic disruption [1]. Recently, the first MARV outbreak recorded in West Africa occurred in Guinea (August 2021), and within less than a year of that outbreak, the second ever MARV outbreak recorded in West Africa occurred in Ghana (July 2022) [4,5]. MARV is reintroduced into the human population through zoonotic transmission and then spread via contact with infectious bodily fluids [1]. Since MARV reservoirs, such as the Egyptian fruit bat (*Rousettus aegyptiacus*), are beyond human control and because human activities persistently encroach on habitats, continued reintroductions of virus into the human population as well as geographic expansion of MARV throughout Africa can be expected [6,7].

There are currently no vaccines licensed for MVD despite the high clinical relevance and more than 50 years of vaccine research since its first identification [1]. There is precedent, however, for licensure of filovirus vaccines; Ebola virus (EBOV) (a filovirus of a different genus but of the same family as MARV) vaccines have been licensed [8,9,10,11,12,13].

Currently licensed EBOV vaccines and many candidate vaccines against other viruses, including MARV, are designed using the backbone of another, less virulent virus as a conduit (vector) to deliver filoviral antigens [8,9,10,11,13,14]. The surface glycoprotein (GP), a target antigen for all these vaccines, is a highly antigenic component of the viral envelope that trimerizes to form spikes at the virion surface that engage with host cell receptors to initiate viral entry [15,16]. Following immunization, MARV vaccine infects the cells using the genetic machinery of the viral vector, delivering the GP gene and ultimately triggering antigen expression and MARV GP-specific humoral and cell-mediated immunity.

Among the candidate MARV vaccines that have been tested in animal studies, there are at least two that have also been tested in human clinical trials [17,18,19]. One of these is a replication deficient chimpanzee adenovirus type 3 (ChAd3)-vectored vaccine that we discuss below. Several preclinical candidate MARV vaccines have been tested in nonhuman primates (NHP), which are considered the gold standard for filovirus vaccine pre-clinical efficacy testing because disease pathophysiology compared to humans is similar [20,21,22,23,24,25,26,27,28,29,30]. Typically, immunogenicity, safety and efficacy are demonstrated in NHPs prior to moving to human clinical trials.

Here, we present NHP data in crab-eating macaques (cynomolgus macaques) for the ChAd3-based vaccine expressing MARV Angola GP. This ChAd3 platform has low pre-existing seroprevalence against the vector, and a good safety profile in humans [31,32,33,34,35]. In NHPs, this vaccine platform has shown strong protection and durable immunity against EBOV and MARV, both with single shot administration against lethal virus challenge [25,36].

The Sabin Vaccine Institute (Sabin) has continued development of the ChAd3-MARV vaccine, originally developed by the National Institutes of Health Vaccine Research Center (NIH VRC) and Glaxo Smith Kline Biologicals SA (GSK), with the intent of pursuing vaccine licensure through the United States (U.S.) Food and Drug Administration (FDA) Animal Rule pathway [37]. In this study, we show that a single shot of ChAd3-MARV vaccine, manufactured in a new formulation intended to enhance vaccine stability, protected NHPs from lethal MARV challenge. We used a qualified anti-MARV GP IgG ELISA that will be needed to support regulatory review by immunobridging between macaques and humans to assess pre-vaccination immune responses.

These data confirm the robustness of protection induced by the ChAd3-MARV vaccine in NHPs and indicate that efficacy is maintained in this new formulation. These results also demonstrate the reproducibility of vaccine-induced protection conferred by ChAd3-MARV as all work was performed in a different Biosafety Level 4 facility than where the original ChAd3-MARV NHP studies were done, and a different team performed the study [25]. This work lays the foundation for future studies with this new vaccine formulation which will be used to establish an immune correlate of protection in pursuit of vaccine licensure via the Animal Rule.

## 2. Materials and Methods

### 2.1. Vaccine Production and Formulation

The ChAd3-MARV (Angola) GP vaccine product for this study was produced at ReiThera, srL, Rome, Italy based on an earlier process developed and completed at Advent, srL, Pomezia, Italy for previous non-clinical and clinical studies. The new formulation produced at ReiThera srL differs from the Advent-produced formulation in the absence of ethanol and Ethylenediaminetetraacetic acid (EDTA), and it contains higher sucrose and lower NaCl concentrations. These were removed with the intention of improving the stability of the formulation at higher storage temperatures.

Briefly, in order to produce the ChAd3 Marburg Angola Drug Substance (DS) (Lot N. RL 20-0004), Procell 92.S Master Cell Bank (Lot No.: A.0005) was thawed and amplified until infection. The amplified cells were infected with Master Virus Seed C.0004 in a 25 L Wavebag and further amplified until harvest. The harvest was performed 72 h post infection. The bulk harvest was subjected to lysis by using a lysis buffer containing 0.2% polysorbate-20. Post lysis the harvest was subjected to Depth Filtration by using one D0HC 0.55 m^2^ filter followed by concentration, diafiltration via Tangential Flow Filtration.

Subsequently, purification and formulation of ChAd3 Marburg Angola DS was completed by SartoQ Anion Exchange Chromatography and a polishing step by IEX Chromatography in flow through mode. The eluate was concentrated and subjected to enzymatic digestion using Benzonase to reduce the host cell DNA amount. Final formulation by Tangential Flow Filtration and diafiltration into A195 Light buffer followed, and the purified material was sterilized using a 0.22 µm filter. The DS was bottled and frozen until subsequent thawing for drug product formulation and filling as described below.

The Drug Product (DP) aseptic manufacturing process included the thawing and dilution of ChAd3 Marburg Angola DS, sterile filtration and dispensing of the ChAd3 Marburg Angola Drug Product into vials followed by an automated vial capping and crimping procedure and subsequent 100% visual inspection. The formulation buffer (A195 Light Buffer) used in both vaccine DS and DP is composed of 10 mM Tris, 10 mM Histidine, 8% Sucrose (*w*/*v*), 25 mM Sodium Chloride, 1 mM Magnesium Chloride, 0.02% Polysorbate 80 (PS-80) (*w*/*v*), pH 7.4. This formulation was selected for improved stability of the materials when stored at either ≤−60 °C or −20 ± 10 °C.

The ChAd3-Marburg vaccine candidate consists of DP ChAd3 Marburg Angola, lot number (LN) RL20-0006, and is a sterile, non-adjuvanted, buffered, aqueous solution consisting of the ChAd3-Marburg (LN RL20-0004) drug substance that is filled into single-dose vials. Each vial contains 1.2 ± 0.1 mL volume of vaccine at a total concentration of 8.21 × 10^10^ virus particles (vp)/mL in formulation buffer.

### 2.2. Animal Study

Texas Biomedical Research Institute is accredited by the Association for Assessment and Accreditation of Laboratory Animal Care, and animal research was conducted under an Institutional Animal Care and Use Committee (IACUC)-approved protocol (1747MF). An intravenous overdose of sodium pentobarbital was used for all euthanasia, and a veterinarian approved all euthanasia. To minimize pain and distress, euthanasia criteria were developed [38,39,40].

Animals were housed at Animal Biosafety Level 2 (ABSL-2) for vaccination, and then underwent a 1-week acclimation period at Animal Biosafety Level 4 (ABSL-4), prior to challenge. Animals were fed a certified primate diet from Purina Mills (Diet 5048), water was available ad libitum, and inanimate enrichment and food enrichment were provided. Excreta pans under the cages, cage flooring, and room floors were cleaned daily. Targeted environmental conditions were a temperature of 74 °F ± 10 °F and relative humidity of approximately 30 to 70%. The light cycle was approximately 12 h on/12 h off. Animals were observed at least twice daily. Clinical observations evaluated 13 different parameters (previously described) [40]. Animals were observed more frequently, three or four times a day, as clinical signs warranted.

**Figure 1:**
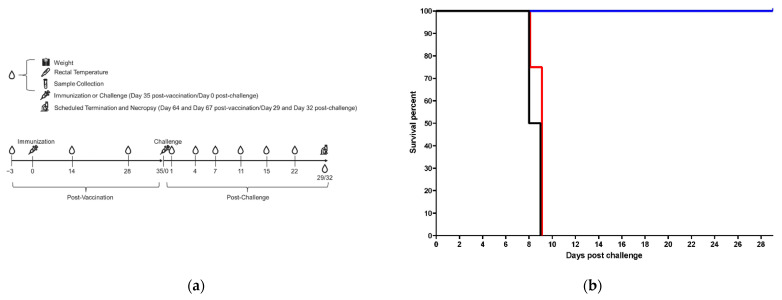

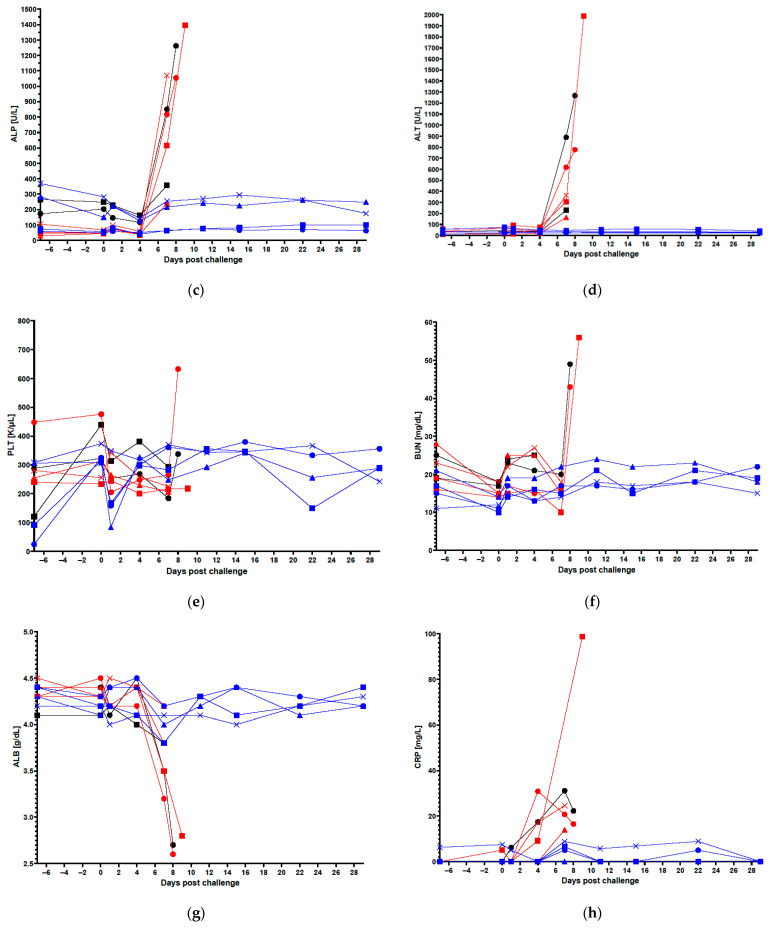

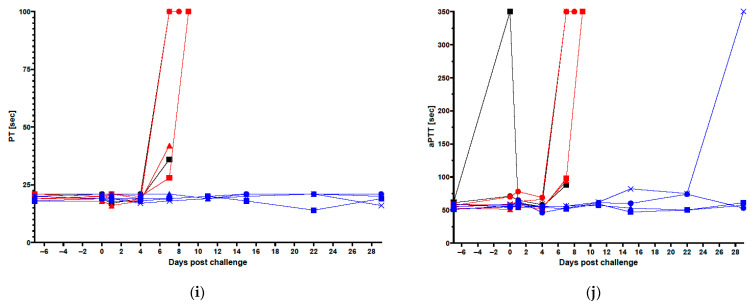
Experimental design, post-challenge survival results and clinical pathology results for control and vaccinated animals. (**a**) experimental design, immunization and bleeding schedule; (**b**) post-challenge survival results; (**c**) ALP, reference interval, 11.34–308.38 U/L; (**d**) ALT, reference interval, 14.16–97.50 U/L; (**e**) PLT, reference interval, 83.48–1009.07 K/µL; (**f**) BUN, reference interval, 7.58–24.40 mg/dL; (**g**) ALB, reference interval, 3.55–5.81 g/dL; (**h**) CRP; (**i**) PT, reference interval, 14.40–27.27 s (sec); (**j**) aPTT, reference interval, 31.12–92.66 s. All reference intervals are the values spanning 95% of the lower bound and 95% of the upper bound. Reference intervals were determined based on healthy rhesus and cynomolgus macaques at Texas Biomedical Institute. 1 × 10^11^ vp animals are shown in blue. 1 × 10^6^ vp animals are shown in red. Saline control animals are shown in black. Survival data are graphed by group using corresponding colors. NHP1 of each group is shown as a circle, NHP2 is shown as a square, NHP3 is shown as a triangle and NHP4 is shown as an “×”. The following animals were found dead in cage (FDIC): NHP4, 1 × 10^6^ vp (FDIC on day 9 post-challenge; last sample collection was day 7 post-challenge) and NHP2, Saline (FDIC on day 9 post-challenge; last sample collection was day 7 post-challenge). Blood was not collected on the day the animals were FDIC. Two animals that survived to the scheduled end of project were euthanized on day 29, and two others were euthanized on day 32. Data for all four surviving animals are depicted on day 29.

Procedure days/study time points were scheduled, as described in Figure 1a. Animals were immunized on day 0 post-vaccination; they were challenged on day 35 post-vaccination (equivalent to day 0 post-challenge). On each post-vaccination procedure day, −3, 14 and 28, blood was collected for complete blood cell counts, serum chemistry analysis, coagulation analysis and immunoassays. On day 35 post-vaccination, blood was collected prior to virus exposure for viral load analysis by quantitative reverse transcription polymerase chain reaction (qRT-PCR) and plaque assay as well as for complete blood cell counts, serum chemistry analysis, coagulation analysis. Blood was collected on all post-challenge procedure days/time points, scheduled and unscheduled (unscheduled procedures days are those on which animals met euthanasia criteria), for complete blood cell counts, serum chemistry analysis, coagulation analysis and viral load analysis by qRT-PCR and plaque assay. If an animal was found dead in its cage, blood was not collected. Rectal temperatures and body weights were recorded for all animals (while sedated) on each procedure day, scheduled and unscheduled. Tissues were harvested at scheduled study termination (days 29 and 32 post-challenge) as well as on unscheduled procedure days. Tissues were also harvested from animals found dead in their cage for histopathological and viral load analysis by qRT-PCR and plaque assay. All methods are described further below (Section 2.3, Section 2.4, Section 2.5, Section 2.6 and Section 2.7).

### 2.3. Vaccine Material and Control Preparation

Vaccine material was provided in single-dose vials (8.21 × 10^10^ vp/mL in 1.2 mL vial volume). Placebo control was sterile saline (069169; Covetrus, Dublin, OH, USA). Control material was handled first, and the appropriate number of 1 mL syringes (309628; BD, Franklin Lakes, NJ, USA) were prepared containing control article. An appropriate number of vaccine vials were thawed, pooled and drawn into an appropriate number of 1 mL syringes for the preparation of the 1 × 10^11^ vp vaccination dose. Due to the titer of the vaccine stock (8.21 × 10^10^ vp/mL), 1 × 10^11^ vp vaccination dose preparation was not diluted. For the 1 × 10^6^ vp vaccination dose prep, vaccine vials were thawed, pooled and then diluted in A195 Light formulation buffer diluent to a concentration of 1 × 10^6^ vp/1.2 mL. Material was delivered within 4 h of thawing. Animals were sedated and each given two injections of 0.6 mL in the left and right quadriceps muscle (intramuscular, IM). Injection sites were monitored daily for five days post vaccination to ensure no adverse reactions.

### 2.4. Challenge Virus, Preparation and Back-Titration

The challenge virus stock was derived from a second-cell culture passage of MARV (Homo sapiens-tc/AGO/2005/Angola-0501379) supplied by Dr. Tom Ksiazek at the National Institute of Allergies and Infectious Diseases (NIAID’s) World Reference Center for Emerging Viruses and Arboviruses (WRCEVA) at the University of Texas Medical Branch (UTMB’s) Health Galveston National Laboratory in 2012. This seed virus was propagated in Vero E6 cells, as described previously, to generate a Passage 3 challenge stock [41].

Prior to virus exposure/challenge, NHPs were sedated (via IM injection) with Telazol (tiletamine hydrochloride/zolazepam hydrochloride; Zoetis Inc., Parsippany-Troy Hills, NJ, USA). The MARV Angola challenge stock was diluted to a target concentration of 2000 plaque-forming units (PFU)/mL in sterile Dulbecco’s phosphate-buffered saline (DPBS, 14190144; Gibco, Thermo Fisher Scientific, Waltham, MA, USA), and each animal was exposed in the right deltoid muscle of the arm to a single injection of 0.5 mL of the diluted MARV Angola. Following preparation, an aliquot was removed for Neutral Red Agarose Overlay (NRAO) plaque assay (as previously described), performed by two independent technicians; the back-titer (averaged from the two independently performed assays) was 339 PFU/mL [40]. After challenge, animals were sedated at scheduled time points; rectal temperatures, body weight, and blood were collected at each sedation (Figure 1a).

### 2.5. Blood for Clinical Chemistry, Hematology and Coagulation

Blood was collected from sedated animals at scheduled time points, as described in Section 2.2 and Figure 1a. Coagulation analysis, complete blood counts (CBC), and clinical chemistry analyses were performed, as previously described [38,39,40,41]. Serum was also analyzed for C-Reactive Protein (CRP) levels using a Piccolo BioChemistry Panel Plus on a Vet Scan analyzer (Abaxis, Inc., Union City, CA, USA). Hematology and serum chemistry parameters shown below (Table 1) are provided (Figure 1c–h). The coagulation assessments performed were activated partial thromboplastin time (aPTT) and prothrombin time (PT) (Figure 1i,j).

### 2.6. Serum and Tissue Viral Load Analysis by Plaque Assay and qRT-PCR

Blood collection for serum isolation for viral load analysis by qRT-PCR and plaque assays was performed, as described in Section 2.2 and Figure 1a. Tissues were collected at scheduled study termination, unscheduled euthanasia as well as when animals were found dead in their cage; tissues collected for viral load analysis by qRT-PCR and plaque assay were lung (lower left lobe), spleen, liver, axillary lymph node from the virus-inoculated arm, adrenal gland, and brain. Viral loads in serum and tissues were determined via plaque assay, as previously described [40]. Viral loads were determined by qRT-PCR as described here. Viral RNA was quantified in serum and tissue samples via qRT-PCR. One-step qRT-PCR was performed using RNA UltraSense One-Step Quantitative RT-PCR System (Invitrogen, Carlsbad, CA, USA), with primers and probe designed to detect a region of the MARV glycoprotein (GP) gene. The primer and probe sequences were as follows: *Marburg marburgvirus* Forward Primer: 5′ GGC CTT CAG GGC AGG TGT A 3′; *Marburg marburgvirus* Reverse Primer: 5′ CCT GTG CAT GAG GGT TTT GA 3′; *Marburg marburgvirus* Probe: 6-FAM 5′ CCT TGC TGT TAG ATC CTC CTA CCA A MGB-NFQ-3′.

### 2.7. Histopathology

Necropsies were performed on all animals. Necropsies were performed as soon as possible, but no later than 12 h after euthanasia or an animal being found dead in its cage. Gross pathological and histopathological assessments were conducted. Tissues collected were heart, lung (left and right lobes), spleen, liver, exposure site (including associated skin and muscle), axillary lymph node from the right (virus-inoculated) arm, right inguinal lymph node, mediastinal lymph node, adrenal gland, stomach, duodenum, jejunum, ileum with gut-associated lymphoid tissue (GALT), colon, rectum, eye, testes/ovaries, brain and gross lesions. Gross pathology findings were recorded at necropsy. A board-certified veterinary pathologist conducted all assessments in a blinded fashion.

All tissues were inactivated and fixed in 10% neutral-buffered formalin for at least 14 days. Paraffin-embedded tissue blocks were prepared following trimming and standard processing of tissue. Sections from these blocks were cut in 5 µm sections for slide preparation. All slides were deparaffinized prior to hematoxylin and eosin (H&E) staining.

### 2.8. Anti-MARV GP IgG ELISA

The qualified anti-MARV Glycoprotein (GP) Immunoglobulin G (IgG) Enzyme-Linked Immunosorbent Assay (ELISA) was performed following the same method as described for the validated anti-Ebola Virus (EBOV) GP IgG ELISA, with changes in materials specific for MARV, as described below (Table 2) [42,43]. In summary, 96-well microplates were coated with 0.2 µg/mL (20 ng/well) of a MARV-specific recombinant glycoprotein (rGP, lot 9 January 2021; Integrated Biotherapeutics, Rockville, MD, USA) [44]. A quality control-high (QCH, lot BMIMARV108), quality control-low (QCL, lot BMIMARV109), and negative control (NC, lot 062121-KE001, originating from serum pooled from 8 NHPs pre-vaccination) were used to assess plate performance. The QCH and QCL were created by individually diluting purified human IgG polyclonal anti-MARV antibodies derived from a transchromosomal (Tc) bovine vaccinated with MARV virus-like particle (VLP) (Musoke strain) antigens (IBT BioServices Cat. No. 0566-001, Rockville, MD, USA) (SAB-170M Anti-Marburg Antibody, Lot #RM1801292MV) with naïve human serum at the appropriate dilution for each reagent. The naïve human serum used as the diluent for these MARV-positive lots was lot BMI547. BMI547 is a pool of 19 individual naïve human serum samples from Innovative Research (Novi, MI; Cat. No. IPLA-SERS). The reference standard (RS, lot BMIMARV107, same origin as QCs) was initially diluted 1:105.8 and then serially diluted 1:1.7 to create an 11-point standard curve. Test Samples (TS) and QCs were run at a 1:50 starting dilution and diluted two-fold down the plate to create a 6-point dilution series. The GP-specific antibodies present in the samples/standards were allowed to bind to the rGP-coated plate. The bound anti-GP IgG antibodies were then detected using a conjugate secondary antibody (horse-radish peroxidase, 1:30,000 dilution, lot 150,445; Jackson ImmunoResearch, West Grove, PA, USA) followed by the addition of 3,3′,5,5′-tetramethylbenzidine (TMB) substrate and stop solution, which induces a color change that is proportional to the anti-GP IgG concentration in the well. Plates were read on a BioTek Epoch microplate reader (BioTek Instruments, Winooski, VT, USA) and the binding concentration of each sample/control was determined in ELISA units/mL.

Following determination of anti-GP IgG concentrations in serum samples, EC_90_ values were determined by calculating a ratio of the positive control (PC) optical density (OD) to the ODs of each test sample dilution. The positive control OD was assigned as 1.88 and was determined by averaging the OD of the QCH at the 1:50 dilution across all plates. The log of the ratio of the sample OD to the positive control OD was plotted on the Y-axis, while the log of the dilution factor at each dilution was plotted on the X-axis. A quadratic equation was fitted to the data, which was then used to back-calculate the dilution at which there was a 90% decrease in antigen binding.

### 2.9. NHP Immunization for PBMC Harvest and ELISpot

In an independent study separate from that described above in Section 2.1, Section 2.2, Section 2.3, Section 2.4, Section 2.5, Section 2.6, Section 2.7 and Section 2.8, male and female Cambodian cynomolgus macaques (ages 3 to 8, weighing 2.8–7.7 kg) were acquired from Envigo Global Services (Alice, TX, USA). This study was performed at Labcorp’s Antibody Reagents and Vaccines Division (formerly of Covance Laboratories, Inc., Danver, PA, USA). Animals were maintained on LabDiet Laboratory Fiber-Plus^®^ Monkey Diet 5049 (PMI Nutrition International, LLC, St. Louis, MO, USA) twice daily in addition to receiving treats, fruits and vegetables daily. Water was provided ad libitum. Toys and enrichment were provided. The room in which animals were housed was maintained at 64–84 °F with 50% (±20) humidity; temperature and humidity were monitored throughout the study using the Kaye LabWatch/Metasys/Johnson Control system. The light cycle was approximately 12 h on/12 h off. Animals were monitored at least twice daily by technical staff; veterinarians provided weekly observations. Animals were sedated for procedures using 12 mg/kg Ketamine (VetOne, Boise, ID, USA) at a concentration of 100 mg/mL and 0.01 mg/kg Dexdomitor (Zoetis, Parsippany-Troy Hills, NJ, USA) at a concentration of 0.5 mg/mL. The study was performed in compliance with the U.S. Department of Agriculture’s (USDA) Animal Welfare Act (9 CFR Parts 1, 2, and 3), the Guide for the Care and Use of Laboratory Animals and the National Institutes of Health, Office of Laboratory Animal Welfare under protocol 0063-20 [45].

Immunizations were performed with ChAd3-MARV or ChAd3-EBO-S (carrying the Sudan virus GP) vaccines that have previously been tested in humans [18,19]. The formulation of these vaccines is similar to the modified formulation described in Section 2.1. All animals received a single dose of ~1 × 10^11^ particle units (PU) of vaccine in 1 mL delivered IM in the right quadricep within 2 h of vaccine thaw on study day 0. Animals were not exposed to Marburg virus or Sudan virus. Blood collections were performed throughout the study on days −7, 14, 28, 42 and 56 post-vaccination. Peripheral blood mononuclear cells (PBMCs) were isolated on days 14 and 56 post-vaccination and frozen (at a concentration of 4–30 × 10^6^ cells/mL, in 1 mL aliquots and target cell viability of >90% in liquid nitrogen). The study was terminated on day 56, and all animals were euthanized.

The ACCUSPIN system (Accuspin-Histopaque-1077, Millipore-Sigma, St. Louis, MO, USA) was used for PBMC isolation. ACCUSPIN tubes were placed at room temperature prior to initiation of isolation. Tubes were centrifuged at 1000× *g* for 1 min at room temperature before use in cases when the Histopaque was not below the frit. Blood (10 mL/tube) was collected in sodium heparin blood collection tubes, and then it was poured into the upper chamber of the ACCUSPIN tube within 2 h of collection. Blood collection tubes were next rinsed with 10 mL PBS (Hyclone Laboratories, Logan, UT, USA), and the rinsed blood and PBS mixture was transferred to the ACCUSPIN tube. Additional PBS was added to the ACCUSPIN tube at a ratio of 1:1, PBS (plus rinse) to blood. Next, the ACCUSPIN tube was centrifuged at 1400× *g* for 30 min at room temperature. The plasma layer was removed to within 0.5 cm of the buffy layer. Plasma was transferred to a glass vial and stored at −80 °C. Then, the remaining volume above the frit was removed from the ACCUSPIN tube and transferred to a 50 mL conical tube. A total of 20–30 mL Ca-Mg free PBS with 2% FBS (Atlas Biologicals, Fort Collins, CO, USA) was added to the conical tube. The conical tube was next centrifuged at 1500 rpm for 10–15 min at room temperature. Following centrifugation, supernatant was aspirated and discarded. Cell pellets from the same animals (in cases where there were multiple tubes for a single animal) were pooled. Pellets were then resuspended in 5–10 mL Ca-Mg free PBS (or RPMI) with 2% FBS. Roughly 50–100 µL of the cell suspension was then removed to determine cell viability via Cellometer Auto 2000 (Nexcelom Bioscience, Lawrence, MA, USA). In the final steps, cells were spun at 1500 rpm for 10 min at room temperature. Supernatant was then aspirated and discarded. Cells were resuspended for freezing in 90% FBS with 10% DMSO (Millipore-Sigma, St. Louis, MO, USA) freezing media. Immediately following, cryovials were first placed in CoolCell (Corning, Corning, NY, USA) (or equivalent) overnight at −80 °C. The following day they were moved to liquid nitrogen.

PBMCs derived from ChAd3-MARV and ChAd3-EBO-S-vaccinated animals above (in this section) were run in an IFNγ ELISpot. ChAd3-EBO-S-vaccinated animals were included as specificity controls. A subset of frozen PBMCs were thawed, washed, and rested overnight in a humidified incubator (37 °C with 5% CO_2_). PBMCs were then washed and 0.2 million cells per well were added to ELISpot^PLUS^ plates pre-coated with anti-IFN-γ antibodies (Cat. No. 3421M-4HPW, Mabtech, Stockholm, Sweden). Triplicate wells were plated for negative control and experimental peptide stimulation conditions. Single wells were plated for positive control stimulation. The cells were stimulated with two MARV peptide libraries (synthesized by Mimotopes, Victoria, Australia), each consisting of 84 15-mer peptides with an 11 amino acid overlap, based on Swiss-Prot ID Q1PD50, which was derived from the human MARV strain Angola/2005 (Genbank, ABE27015.1), at a concentration of 2 µg/mL/peptide for 24 h. Cells were treated with 25 µg/mL phytohemagglutinin (PHA) as a positive control and 0.4% dimethyl sulfoxide (DMSO) as a negative control. Cells and stimuli were removed, and the plate was washed prior to incubation in the detection antibody (7-B6-1-Biotin Cat. No. 3421M-4HPW, Mabtech) for two hours at room temperature. Streptavidin-HRP and then its substrate solution (TMB, Cat. No. 3421M-4HPW, Mabtech) were subsequently added. The reaction was developed until distinct spots appeared, after which it was stopped by extensive washing with water. After air drying, the plate was read using an ImmunoSpot Series 3B ELISpot plate reader (Cellular Technology Limited, Cleveland, OH, USA) with software version 5.1. Data were analyzed by subtracting the mean negative control spot count from the mean of each experimental condition and normalizing the result to spots per 10^6^ PBMCs.

## 3. Results

### 3.1. Clinical Data in Vaccinated and Control Animals

Animals were vaccinated intramuscularly (IM) with 1 × 10^11^ vp ChAd3-MARV (*n* = 4), 1 × 10^6^ vp ChAd3-MARV (*n* = 4) or saline control (*n* = 2) 35 days prior to virus challenge. The 1 × 10^6^ vp- and 1 × 10^11^ vp-vaccinated groups differ only in the dose of the vaccine used for vaccination. Blood was collected on days −3, 14 and 28 post-vaccination (7 days prior to challenge). Blood was also collected on the challenge day (prior to virus exposure) and days 1, 4, 7, 11, 15 and 22 post-challenge as well as at planned study termination and unplanned euthanasia (Figure 1a). On challenge day, animals were exposed to a target dose of 1000 PFU MARV/Angola via IM injection.

All 1 × 10^6^ vp-vaccinated and saline control animals either met euthanasia criteria or succumbed to MARV infection on day 8 or 9 post-challenge, indicating that the 1 × 10^6^ vp dose provided no benefit in delaying time to death/euthanasia. All 1 × 10^11^ vp-vaccinated animals survived (Figure 1b). Body weight loss in all 1 × 10^11^ vp-vaccinated animals (and across all groups) was minimal to undetectable (Figure S1a) when compared to baseline. Two of the four 1 × 10^11^ vp-vaccinated animals gained weight during the critical phase of the study (Figure S1a). Overall, rectal temperatures were variable, but trends were observed post-challenge. Only one of the four 1 × 10^11^ vp-vaccinated animals recorded a rectal temperature above 2 degrees Fahrenheit over baseline (the average of the rectal temperature recorded on day 28 post-vaccination and day 35 post-vaccination/day 0 post-challenge); this occurred on day 1 post-challenge, again on day 22 post-challenge and at scheduled study termination. It should be noted, however, that this animal recorded the lowest average baseline temperature among all animals and also recorded variable rectal temperatures in the post-vaccination phase of the study. Thus, natural temperature fluctuation in this animal cannot be ruled out as a cause for these increases. Most 1 × 10^6^ vp-vaccinated and saline control animals spiked temperatures 2 degrees or more above baseline on at least one day post-challenge before temperatures declined by ~10 degrees or more in some animals as animal condition worsened (Figure S1b). No 1 × 10^11^ vp-vaccinated animal recorded a post-challenge rectal temperature above 100.9 °F, whereas all 1 × 10^6^ vp-vaccinated and saline control animals did on at least one day. Clinical scores did not exceed 4 (a score of ≥15 required euthanasia) in any 1 × 10^11^ vp-vaccinated animals post-challenge; all 1 × 10^6^ vp and saline control animals recorded post-challenge clinical scores at least as high as 7 but reaching as high as 39 (Figure S1c). Overall, there was a clear difference in the condition and health of animals in the 1 × 10^11^ vp-vaccinated group compared to the other groups.

Complete blood cell counts (CBCs) and clinical chemistries were evaluated throughout the study. Coagulation tests were also performed. Here, we focus on key analytes in post-challenge samples to assess liver damage, kidney function and coagulopathy, all important in MARV disease pathogenesis [2,46,47,48,49]. Analytes included alkaline phosphatase (ALP, a liver enzyme), alanine transaminase (ALT, a liver enzyme), platelets (PLT, a blood cell fragment involved in clotting), blood urea nitrogen (BUN, an indicator of kidney function and hydration), albumin (ALB, a liver protein that informs on liver and kidney function), c-reactive protein (CRP, a liver protein indicator of inflammation), prothrombin time (PT, an assessment of coagulation) and activated partial thromboplastin time (aPTT, an assessment of coagulation) (Figure 1c–i).

Increases in ALP and ALT were observed mostly by day 7 post-challenge indicating liver damage in the 1 × 10^6^ vp-vaccinated and saline control groups; values in 1 × 10^11^ vp-vaccinated animals remained relatively low and consistent (Figure 1c,d). In contrast, PLT values were variable in all groups with all values within the expected normal range (Figure 1e). BUN values remained relatively steady post-challenge in the 1 × 10^11^ vp-vaccinated group; all values were within the expected normal range. BUN values in some 1 × 10^6^ vp-vaccinated and saline control animals increased, consistent with decreased glomerular filtration rate (GFR). Notably, the animals that registered elevated BUN values were those that met euthanasia criteria on the same day (days 8 and 9 post-challenge); other animals in those groups were found dead on days 8 and 9 post-challenge prohibiting the collection of samples (Figure 1f). Similar to the BUN value findings, ALB values remained steady in all groups until about day 7 post-challenge when declines were recorded; 1 × 10^6^ vp-vaccinated and saline control animals that met euthanasia criteria on days 8 and 9 post-challenge registered decreases in ALB below the normal range (Figure 1g). ALB values in the 1 × 10^11^ vp-vaccinated group remained steady on all days assayed and were within the expected range for healthy animals. The days these changes were recorded are consistent with other MARV NHP studies [25,48,49]. CRP values were variable and frequently hovered around the limit of detection of the assay for all groups, but by day 4 post-challenge, several animals in the 1 × 10^6^ vp-vaccinated and saline control groups had CRP values that appeared to represent real increases as they were followed by consecutive elevated CRP values on subsequent collection days (Figure 1h). Other MARV studies have shown CRP increases in infected untreated animals on roughly the same day [25,48,49]. Finally, the examination of coagulation assessments, PT and aPTT, indicated coagulopathy (Figure 1i,j). Increased PT and aPTT, which were noted on day 7 post-challenge in the 1 × 10^6^ vp-vaccinated and saline control groups, suggest the blood clotting was adversely affected by MARV infection as expected [47,49]. Importantly, 1 × 10^11^ vp-vaccinated animals blood coagulation time generally remained steady throughout the post-challenge phase of the study and were within the normal expected range. A spike in aPTT was recorded in NHP4, 1 × 10^11^ vp on the final day of the study (at scheduled study endpoint); however, a similar spike was observed on challenge day for NHP2, saline. The spike in the saline animal was transient. Transient spikes can occur in healthy animals and could also be due to sample collection (i.e., blood may have started to clot at the time of assessment).

Overall, these findings in the 1 × 10^6^ vp-vaccinated and saline control groups were consistent with MARV-induced changes observed in other NHP studies and in infected humans [2,25,47,48]. Clinical values for the critical analytes were virtually indistinguishable between the 1 × 10^6^ vp-vaccinated and saline control animals, suggesting no protective effect at the 1 × 10^6^ vp dose. Conversely, the 1 × 10^11^ vp-vaccinated animals exhibited few, if any, of the stereotypical MARV clinical pathologies on the days assayed.

### 3.2. Post-Challenge Serum Viral Loads by qRT-PCR and Plaque Assay

**Figure 2:**
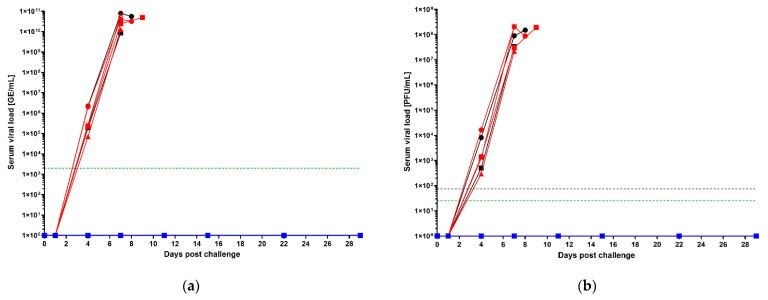
Post-challenge viremia. (**a**) serum viral loads by qRT-PCR; (**b**) serum viral loads by plaque assay. 1 × 10^11^ vp animals are shown in blue. 1 × 10^6^ vp animals are shown in red. Saline control animals are shown in black. NHP1 of each group is shown as a circle, NHP2 is shown as a square, NHP3 is shown as a triangle and NHP4 is shown as an “×”. Lower limit of detection (LOD) is shown as a green dashed line, and the lower limit of quantitation (LLOQ) is shown as a gray dashed line. In cases where the LOD is also the LLOQ, a single green dashed line is shown. The following animals were found dead in cage (FDIC): NHP4, 1 × 10^6^ vp (FDIC on day 9 post-challenge; last sample collection was day 7 post-challenge) and NHP2, Saline (FDIC on day 9 post-challenge; last sample collection was day 7 post-challenge). Blood was not collected on the day the animals were FDIC. Two animals that survived to the scheduled end of project were euthanized on day 29, and two others were euthanized on day 32. Data for all four surviving animals are depicted on day 29.

Beginning on the day of challenge, viral load was assessed in serum samples in all groups by qRT-PCR and plaque assay on all collection days post-challenge (and all unscheduled euthanasia days) as viremia is an important predictor of survival in filovirus disease (Figure 2) [25,50,51]. qRT-PCR results are reported in total genome equivalents (GE) per milliliter (GE/mL). Serum viral loads were detectable beginning on day 4 post-challenge, although no collections were scheduled for days 2 or 3 post-challenge so viral loads were not assessed on those days (Figure 2a). Viral loads by qRT-PCR were similar in saline control and 1 × 10^6^ vp-vaccinated groups; titers reached upwards of 1 × 10^10^ GE/mL in both saline control and 1 × 10^6^ vp groups. In contrast, viral loads in the 1 × 10^11^ vp-vaccinated group were below the limit of detection by qRT-PCR on all days assayed.

Serum plaque assays results (measured in plaque forming units per mL, PFU/mL), indicative of the presence of live virus, were consistent with the serum qRT-PCR results (Figure 2b). Collection days were identical. Control animals and 1 × 10^6^ vp-vaccinated animals had similar viral loads by plaque assay reaching upwards of 1 × 10^8^ PFU/mL while 1 × 10^11^ vp-vaccinated animals did not have detectable serum viral loads by plaque assay on any day assayed.

In all, reduction in viremia coincided with survival. These data are consistent with the survival and clinical pathology results in that they indicate no apparent benefit of vaccination at the 1 × 10^6^ vp dose compared to saline control as well as a clear benefit of vaccination at the 1 × 10^11^ vp dose.

### 3.3. Tissue Viral Loads by qRT-PCR and Plaque Assay

**Figure 3:**
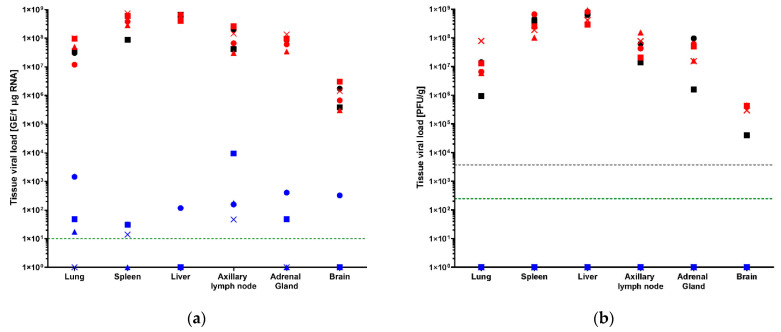
Post-challenge tissue viral loads. (**a**) tissues viral loads by qRT-PCR; (**b**) tissue viral loads by plaque assay. 1 × 10^11^ vp animals are shown in blue. 1 × 10^6^ vp animals are shown in red. Saline control animals are shown in black. NHP1 of each group is shown as a circle, NHP2 is shown as a square, NHP3 is shown as a triangle and NHP4 is shown as an “×”. LOD is shown as a green dashed line, and the LLOQ is shown as a gray dashed line. In cases where the LOD is also the LLOQ, a single green dashed line is shown.

Tissues including axillary lymph nodes, adrenal gland, brain, liver, lung and spleen were collected from all animals at necropsy for viral load analysis by qRT-PCR and plaque assay. Necropsies were performed on all animals regardless of the time point or manner of death. RNA loads were detectable in all saline control and 1 × 10^6^ vp-vaccinated animal tissues at titers exceeding 1 × 10^5^ GE/1 µg RNA in all tissues assayed; titers in most tissues (axillary lymph nodes, adrenal gland, liver, lung and spleen) exceeded 1 × 10^7^ GE/1 µg RNA (Figure 3a). RNA loads in 1 × 10^6^ vp-vaccinated group compared to saline control were similar. Overall, the 1 × 10^11^ vp-vaccinated group showed detectable RNA loads in all tissues assayed; however, not every 1 × 10^11^ vp-vaccinated animal had detectable RNA loads in every tissue assayed. Many tissues had RNA loads near the limit of detection (LOD, 10 GE/1 µg RNA), but there were seven instances when RNA loads exceeded titers of 1 × 10^2^ GE/1 µg RNA, one log above the LOD. Most of these instances showed loads less than 2 logs higher than LOD. Of these seven instances, all but two instances were attributable to the same animal (NHP1 in the 1 × 10^11^ vp group, axillary lymph node, adrenal gland, lung, liver and brain) (Figure 3a). Titers above the LOD were recorded for all 1 × 10^11^ vp-vaccinated animals in the axillary lymph node, but only one 1 × 10^11^ vp-vaccinated animal had RNA load titers above the LOD in the brain. This is the same and only animal that had RNA load titers above LOD in the liver.

Tissue viral loads by plaque assay were consistent with the qRT-PCR results for the 1 × 10^6^ vp-vaccinated and saline control animals in that all animals had viral titers above the LOD (250 PFU/g) in all tissues assayed, confirming the presence of live, replicating virus in those samples (Figure 3b). In contrast, no live, replicating virus was detected in any tissues of any of the 1 × 10^11^ vp-vaccinated animals (Figure 3b). While these assays are not directly comparable, this suggests that the RNA loads were not necessarily the result of live, replicating virus, but they were possibly the result of non-infectious viral genome that had not yet been cleared from those tissue sites (in the case of surviving animals). It should be noted that neither immunohistochemistry nor in situ hybridization were performed to confirm viral infection of any tissues. Viremia may be a contributing component in the viral load titers as no tissues were perfused prior to fixation.

### 3.4. Histopathological Findings in Select Tissues

All animals were necropsied regardless of the time and manner of death. At necropsy, gross findings were recorded. Gross findings attributed to filoviral infection were noted in all the 1 × 10^6^ vp-vaccinated and saline control animals. These findings included, but were not limited to, skin rash, enlarged lymph nodes (correlating with necrosis or hemorrhage), pale liver (correlating with necrosis) and pale spleen (correlating with fibrin). All animals in both groups had gross findings in the liver. In contrast, none of the 1 × 10^11^ vp-vaccinated animals had any gross findings considered to be related to filoviral infection.

In addition to examining gross findings at necropsy, histologic findings were recorded after tissue fixation and H&E staining. A board-certified veterinary pathologist examined the H&E-stained slides in a blinded fashion and rated the severity of the findings. A score of “0” indicated no findings, a score of “1” indicated minimal findings, a score of “2” indicated mild findings, a score of “3” indicated moderate findings, a score of “4” indicated marked findings and a score of “5” indicated severe findings. When findings were present (but ungraded), a “P” was assigned. Consistent with survival, clinical pathology and viral load analyses, no 1 × 10^11^ vp-vaccinated animals had any histologic findings scoring above mild. In contrast, 1 × 10^6^ vp-vaccinated and saline control animals had histologic scores as high as 4. Overall, findings in the 1 × 10^6^ vp-vaccinated group and the saline control animals were similar, and they were consistent with acute filoviral infection corresponding to the similarities observed in survival and viremia between these groups [49]. Major MARV target organs, liver, lymph node and spleen, were affected as anticipated based on serum chemistry findings and tissue viral loads (Figure 1 and Figure 3). MARV-related microscopic findings consistently noted in multiple tissues from the 1 × 10^6^ vp-vaccinated and saline control group animals included necrosis, inflammation, hemorrhage, thrombosis, fibrin, and lymphoid depletion. Summary findings for the challenge site, adrenal gland, brain, liver, lymph node and spleen are shown for all animals for comparison to viral load tissue data (Table 3). No MARV-related findings were observed in the lungs of any animals in any group, consistent with the intramuscular route of exposure [49]. NHP1 dosed with 1 × 10^11^ vp had similar histologic findings compared to other 1 × 10^11^ vp-vaccinated animals despite having detectable viral loads in all tissues by qRT-PCR, loads that were higher than those detected in some tissues of other animals in the same group. Histologic findings in the brain were not noted in any animals in any group, except for NHP2 dosed with 1 × 10^6^ vp, despite the viral loads detected in all 1 × 10^6^ vp-vaccinated and saline control animals as well as NHP1 dosed with 1 × 10^11^ vp.

### 3.5. Anti-MARV GP IgG ELISA Results in Vaccinated and Control Groups

**Figure 4:**
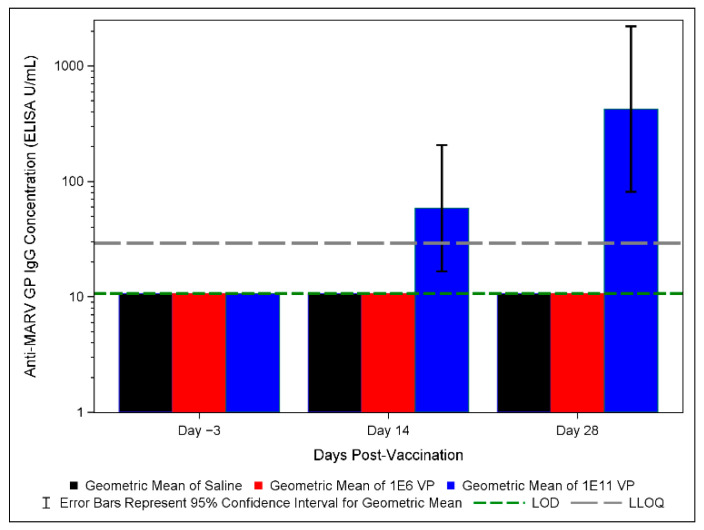
Post-vaccination anti-MARV GP IgG concentrations. Geometric means for each group at day −3, 14 and 28 post-vaccination are graphed. 1 × 10^11^ vp group is shown in blue. 1 × 10^6^ vp group is shown in red. Saline control group is shown in black. LOD is shown as a green dashed line, and the LLOQ is shown as a gray dashed line. Error bars represent 95% confidence internal for the geometric mean.

Serum from all animals was collected prior to challenge on days −3, 14 and 28 post-vaccination for immunological analysis by anti-MARV GP IgG ELISA. We focused on IgG ELISA due to the strong correlation between survival and anti-filovirus GP-binding IgG titer and concentration previously shown; we anticipate that anti-MARV GP-binding IgG concentration will be a correlate of protection for future FDA Animal Rule approval [14,25]. Total anti-MARV GP-binding IgG concentrations were determined based on an assay qualified by Sabin per U.S. FDA guidelines. Results are reported in ELISA units/mL (EU/mL). All surviving (1 × 10^11^ vp-vaccinated) animals had detectable anti-MARV IgG concentrations by day 14 post-vaccination, the earliest post-vaccination time point assayed (Figure 4). None of the 1 × 10^6^ vp-vaccinated or saline control animals had IgG concentrations above the limit of detection. IgG concentrations in the 1 × 10^11^ vp-vaccinated group were variable, ranging from about 109 to 1290 ELISA U/mL; therefore, a wide range of IgG concentrations corresponded to survival. Despite NHP4, 1 × 10^11^ vp, having the lowest IgG concentration, results presented above do not indicate that this animal performed discernably differently than other animals in the same group with higher IgG concentrations.

To contextualize our IgG results, we converted IgG concentrations to EC_90_ titers as described above, allowing a rough comparison to IgG results reported in Hunegnaw et al. in ChAd3-MARV-vaccinated NHPs [25]. On day 28 post-vaccination, the day most proximate to virus challenge, EC_90_ converted titers ranged from 332 to 2448. These values fall within the range reported by Hunegnaw et al. to associate with robust protection in macaques [25]. It should be noted, however, that the ELISA employed by Hunegnaw et al. is distinct from that used in this study, meaning that these data are not quantitatively comparable even after EC_90_ conversion.

Since none of the 1 × 10^6^ vp-vaccinated animals had detectable IgG concentrations and all the 1 × 10^11^ vp-vaccinated animals survived challenge, we were unable to project even a rough correlation between IgG concentration and survival. Still, our data suggest a correlation between survival and IgG concentrations given that only surviving animals had detectable concentrations.

### 3.6. MARV GP-Specific IFNγ Responses in Vaccinated Animals

In a separate experiment from the challenge study described above in Figure 1, Figure 2, Figure 3 and Figure 4 and Table 1, Table 2 and Table 3, cynomolgus macaques were administered a single shot of an approximately 1 × 10^11^ PU ChAd3-MARV or a ChAd3-EBO-S (carrying the GP of Sudan virus) vaccine. ChAd3-EBO-S-vaccinated animals are included here as specificity controls. The vaccine formulation was similar to the formulation used above (Figure 1, Figure 2, Figure 3 and Figure 4 and Table 1, Table 2 and Table 3). Animals were not exposed to virus. PBMCs were harvested and frozen on days 14 and 56 post-vaccination. Thawed PBMCs were used to perform a MARV-specific IFNγ ELISpot assay. Two MARV GP peptide pools were included covering the length of the GP peptide. IFNγ was selected as an indicator of the T cell response, a possible mechanistic factor in protection against filoviral infection [26,52]. Phytohemagglutinin (PHA) was included as a positive control, and DMSO was included as a negative control.

**Figure 5:**
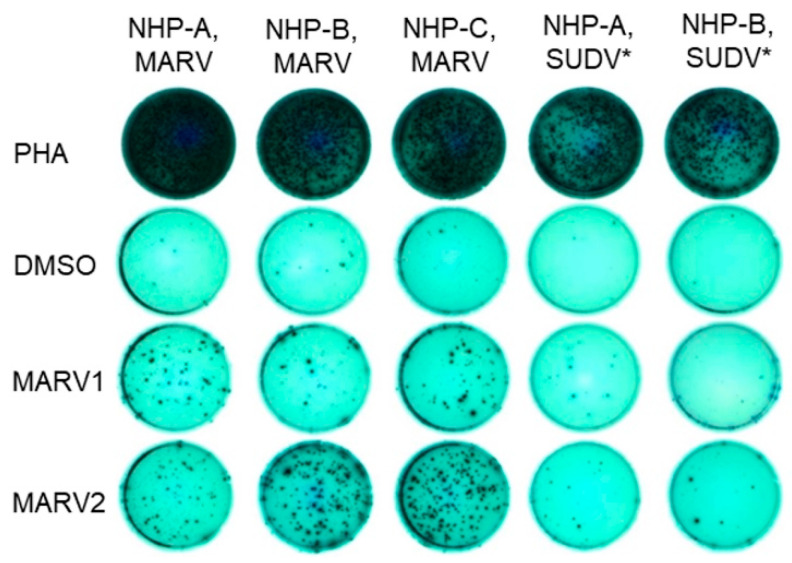
Post-vaccination PBMCs IFNγ ELISpot responses, plate image. Representative IFNγ ELISpot images of PBMCs from Day 56 vaccinated animals incubated with MARV peptide pools as the specific stimuli, DMSO as the negative control, and Phytohemagglutinin (PHA) as the positive control. * PBMC samples collected 14 days post-ChAd3-EBO-S (SUDV) vaccination were included as specificity controls. NHPA-C, MARV animals were vaccinated with ~1 × 10^11^ PU ChAd3-MARV vaccine; NHPA-B, SUDV animals were vaccinated with ~1 × 10^11^ PU ChAd3-EBO-S vaccine. MARV1: MARV peptide pool 1; MARV2: MARV peptide pool 2.

Although a different formulation and experiment than the challenge study, we include these data to demonstrate that the ChAd3-MARV vaccine induced a robust, MARV-specific IFNγ response (Figure 5 and Table 4). Overall, all ChAd3-MARV-vaccinated animals (in response to at least one of the peptide pools) clearly showed increased spot counts over the ChAd3-EBO-S-vaccinated animals (Table 4). All animals demonstrated a robust response to PHA and low background (DMSO condition) (Figure 5). These data suggest that ChAd3-MARVvaccination primed GP antigen-specific T cells that were activated to produce the Th1 cytokine IFNγ upon re-stimulation in vitro.

## 4. Discussion

We have shown that ChAd3-MARV confers 100% protection in NHPs with a single 1 × 10^11^ vp dose against a lethal MARV Angola challenge. Surviving animals showed minimal to no changes in key clinical pathology analytes, body weight, and rectal temperatures. In these animals, clinical scores remained low throughout the study. Viremia (serum viral load) was undetectable by qRT-PCR and plaque assay, respectively, on all days examined. Tissue viral loads were generally low, and histopathology showed only mild findings. All surviving (1 × 10^11^ vp-vaccinated) animals had detectable anti-MARV IgG concentrations by day 14 post-vaccination, while none of the 1 × 10^6^ vp-vaccinated or saline control animals had IgG concentrations above the limit of detection. These results are consistent with the prior observation that anti-GP IgG correlates with survival in ChAd3-MARV-vaccinated NHPs [25].

Our findings are consistent with Hunegnaw et al. overall, and indicate that, although the vaccine formulation differs from that study, the 1 × 10^6^ vp dose offers minimal or no protective effect. Hunegnaw et al. reported survival of just one of four NHPs following vaccination with 1 × 10^6^ particle units [25]. The difference between zero of four and one of four is not statistically significant [25]. Hunegnaw et al. also reported 100% protection at doses of 1 × 10^10^ and 1 × 10^9^ PU with a single shot of ChAd3-MARV prepared in a different formulation buffer than we have used here. Although our dose of 1 × 10^11^ vp is not directly comparable to PU (the former uses qPCR and the latter high-performance liquid chromatography, HPLC, to enumerate virus particles), we did achieve 100% protection with a single dose. We also achieved anti-MARV GP IgG results that, when converted to EC_90_, fell within the range of protective titers identified in their work. In establishing these consistencies, we have shown that the new formulation buffer does not appear to impact efficacy and that ChAd3-MARV vaccine results are reproducible in a different BSL4 laboratory, demonstrating robust protection. Based on these consistencies, we expect the ChAd3-MARV vaccine in A195 Light buffer formulation to induce T cell responses as suggested by the IFNγ ELISpot data herein, and we anticipate the ChAd3-MARV vaccine in the A195 Light formulation to induce both humoral (as we have shown above) and cellular immunity as published for similar vaccines on the ChAd3 platform in NHPs and humans [32,36].

While there are competing MARV vaccine candidates under development, ChAd3-MARV has already shown safety and immunogenicity in humans [19,53]. The ChAd3 platform has been administered to over 5000 individuals with safety shown in adults and children for other filovirus indications [17,18,19,31,32,33,54]. It is also a vaccine platform primed for emergency use deployment. GMP grade vials of ChAd3-MARV drug product are available to be dispatched in an outbreak scenario. We have successfully improved scalability of our new formulation which better positions the vaccine for scaled up production during an outbreak and for stockpiling purposes. Other MARV-specific vaccine platforms have yet to enter clinical trials, require more than one dose to achieve 100% protection in NHPs (exceptions include vesicular stomatitis virus (VSV)-vectored platforms) and to our knowledge do not have GMP doses available [22,23,24,27,28,29,30,53]. Additionally, rapid protection, as early as 1-week post-vaccination, and durable protection, as long as a year post-vaccination, have been demonstrated for ChAd3-MARV in NHPs [25]. While the VSV platforms have documented rapid and durable protection in NHPs, durable protection (to the best knowledge of these authors) has only been published against MARV Musoke, a less virulent strain than MARV Angola, against which ChAd3-MARV has shown durable protection [24,48,53,55]. As of this drafting, the ChAd3-MARV vaccine is the most advanced MARV vaccine in development. It is advanced in clinical trial history, manufacturing (GMP dose availability) and in the assays used to characterize its activity, including a fully qualified anti-MARV IgG ELISA.

This work confirmed our target dose range for dose ranging studies which will be performed between 1 × 10^6^ and 1 × 10^11^ vp. Future work involving dosing studies with the ChAd3-MARV vaccine in our modified formulation will seek to establish a quantitative immune correlation between survival and concentration of GP-binding IgG, and perhaps other immunological parameters. Of note, Hunegnaw et al. have reported that neutralizing antibody titers do not correlate with survival and their results indicate that the concentration of GP-binding IgG is the immune correlate of survival for ChAd3-MARV [25]. As the development progresses, validation of the anti-MARV GP ELISA (and any other assays to be used to establish correlates of protection) will be completed. We will also aim to demonstrate enhanced stability of the ChAd3-MARV vaccine in A195 Light buffer formulation at higher temperatures that improve cold chain logistics and permit storage in facilities where ultralow freezers are not available. Much remains to be achieved, but the robustness of protection and the reproducibility of these results offer promise of a MARV vaccine that will achieve licensure, finally reaching those in affected countries and addressing a long-standing unmet need.

## Figures and Tables

**Table 1:** Hematology and Serum Chemistry Parameters.

Hematology		
Parameter	Abbreviation	Units
Platelet Count	PLT/PCT	K/µL, %
**Serum Chemistry Parameters**		
**Parameter**	**Abbreviation**	**Units**
Alanine Aminotransferase	ALT	U/L
Albumin	ALB	g/dL
Alkaline Phosphatase	ALP	U/L
Blood Urea Nitrogen	BUN	mg/dL
C-Reactive Protein	CRP	mg/L

**Table 2:** Anti-MARV GP IgG ELISA Critical Reagents.

Reagent	Virus/Variant	Supplier	Lot Number	Coating Amount per Well
rGP	MARV/Angola	Integrated Biotherapeutics Rockville, MD, USA	9 January 2021	20 ng
**Reagent**	**Lot Number**	**Supplier**	**Concentration (ELISA Units/mL)**	**Starting Dilution on Plate**
Reference Standard	BMIMARV107	Battelle	1058	1:105.8
Quality Control High	BMIMARV108	Battelle	427.58	1:50
Quality Control Low	BMIMARV109	Battelle	156.09	1:50
Negative Control	062121-KE001	Battelle	0.00	1:50
**Reagent**	**Lot Number**	**Catalog Number**	**Supplier**	**Dilution on Plate**
Conjugate	150,445	109-035-098	Jackson ImmunoResearch West Grove, PA, USA	1:30,000

**Table 3:** Histopathological findings and scores for key tissues in vaccinated and control animals.

Selected Tissue Histopathological Findings											
Site/Tissue	Finding	NHP1, 1 × 10^11^ vp *	NHP2, 1 × 10^11^ vp *	NHP3, 1 × 10^11^ vp *	NHP4, 1 × 10^11^ vp *	NHP1, 1 × 10^6^ vp	NHP2, 1 × 10^6^ vp	NHP3, 1 × 10^6^ vp	NHP4, 1 × 10^6^ vp	NHP1, Saline Control	NHP2, Saline Control
CHALLENGE SITE	Inflammation	0	0	0	0	2	0	2	2	2	0
	Necrosis	0	0	0	0	0	0	2	1	0	0
	Inflammatory cell infiltration	2	0	0	2	0	0	0	0	0	2
	Hemorrhage	0	0	0	0	P	0	0	0	0	0
	Edema	0	0	0	0	P	0	P	0	0	0
	Thrombosis	0	0	0	0	0	0	0	0	0	P
LYMPH NODE	Lymphoid depletion	2	2	0	1	4	4	3	4	4	4
	Lymphocytolysis	0	0	0	0	3	2	1	2	2	2
	Inflammation	0	0	0	0	3	3	0	3	3	0
	Necrosis	0	0	0	0	4	4	2	3	4	4
	Fibrin deposition	0	0	0	0	P	P	0	P	P	P
	Hemorrhage	0	0	0	0	P	P	0	P	P	P
	Sinus histiocytosis	0	2	0	0	2	0	0	0	0	0
	Paracortical hyperplasia	0	0	0	0	0	2	1	0	0	0
	Follicular hyperplasia	1	0	0	0	0	0	0	0	0	0
SPLEEN	Lymphoid depletion	0	2	0	0	3	4	3	3	3	4
	Lymphocytolysis	0	0	0	0	2	1	1	1	2	1
	Fibrin deposition	0	0	0	0	P	P	P	P	P	P
	Congestion/hemorrhage	0	0	0	0	0	P	P	P	P	P
	Follicular hyperplasia	0	0	1	2	0	0	0	0	0	0
LIVER	Necrosis, hepatocellular	0	1	0	0	2	3	2	2	2	1
	Inflammation	0	1	0	0	1	1	1	1	0	1
ADRENAL	Necrosis	0	0	0	0	0	1	0	1	0	0
	Inflammation	0	0	0	0	0	1	0	1	0	0
KIDNEY	Thrombosis	0	0	0	0	0	P	0	0	0	0
BRAIN, CEREBRUM	Hemorrhage, choroid plexus	0	0	0	0	0	P	0	0	0	0
	Thrombosis, choroid plexus	0	0	0	0	0	P	0	0	0	0

1—Minimal; 2—Mild; 3—Moderate; 4—Marked; P—Ungraded finding present; 0—Finding not present; *—Terminal euthanasia (survivor); No findings were observed in the lungs of any animals in any group.

**Table 4:** Post-vaccination PBMCs IFNγ ELISpot responses, spot counts.

Animal ID	Days Post-ChAd3-MARV Vaccination	Mean Spot-Forming Cell Counts/1 × 10^6^ PBMCs	
		MARV Peptide Pool 1	MARV Peptide Pool 2
NHP-A, MARV	56	305	363
NHP-B, MARV		53	868
NHP-C, MARV		188	818
NHP-A, SUDV	N/A	27	60
NHP-B, SUDV		28	32

N/A: not applicable. NHPA-C, MARV animals were vaccinated with ~1 × 10^11^ PU ChAd3-MARV vaccine; NHPA-B, SUDV animals were vaccinated with ~1 × 10^11^ PU ChAd3-EBO-S vaccine. Mean spot-forming counts are reported per 1 × 10^6^ PBMCs.

## Data Availability

The data associated with the findings herein described can be found within the above article and the Supplementary Material.

## Supplementary Materials

The following supporting information can be downloaded at: https://www.mdpi.com/article/10.3390/vaccines10111935/s1, Figure S1: Body weight, rectal temperatures and clinical scores.
