# Supplementary material for: Single-Shot ChAd3-MARV Vaccine in Modified Formulation Buffer Shows 100% Protection of NHPs
Source: Vaccines (Basel). 2022 Nov 15;10(11):1935. doi: 10.3390/vaccines10111935 (PMC9694189; doi:10.3390/vaccines10111935)
Supplement: Supplementary file 1 [file vaccines-10-01935-s001.zip › Figure S1_Body weight, rectal temperatures and clinical scores_11.13.22_revised.pdf]

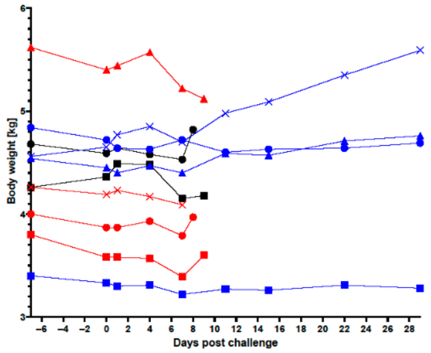

(**a**)

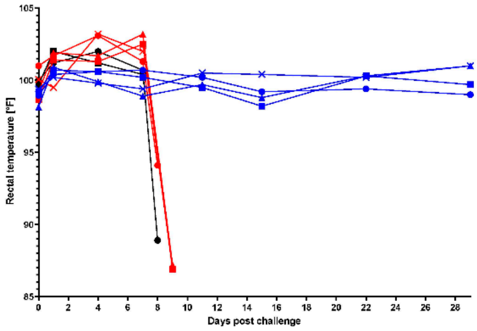

(**b**)

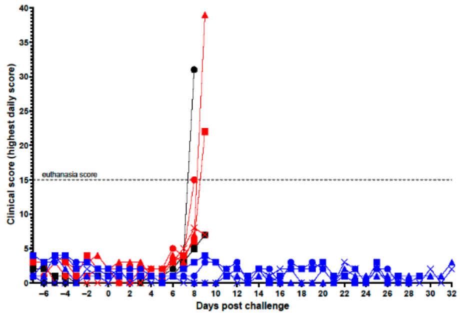

(**c**)

Figure S1. Body weight, rectal temperatures and clinical scores (**a**) body weight; (**b**) rectal temperature from baseline (average of pre-challenge day 28 and day 0 challenge; baseline is shown as day 0); (**c**) clinical scores. 1E11 vp animals are shown in blue. 1E6 vp animals are shown in red. Saline control animals are shown in black. NHP1 of each group is shown as a circle, NHP2 is shown as a square, NHP3 is shown as a triangle and NHP4 is shown as an "x". Two animals that survived to the scheduled end of project were euthanized on day 29, and two others were euthanized on day 32. Data for all four surviving animals are depicted on day 29 except in the case of clinical scores (c).
